# Associations between body-mass index and COVID-19 severity in 6·9 million people in England: a prospective, community-based, cohort study

**DOI:** 10.1016/S2213-8587(21)00089-9

**Published:** 2021-06

**Authors:** Min Gao, Carmen Piernas, Nerys M Astbury, Julia Hippisley-Cox, Stephen O'Rahilly, Paul Aveyard, Susan A Jebb

**Affiliations:** a University of Oxford, Nuffield Department of Primary Care Health Sciences, Radcliffe Observatory Quarter, Oxford, UK; b School of Public Health, Peking University Health Science Center, Beijing, China; c NIHR Oxford Biomedical Research Centre, Oxford University Hospitals, NHS Foundation Trust, Oxford, UK; d University of Cambridge, MRC Metabolic Diseases Unit, Wellcome-MRC, Institute of Metabolic Science and NIHR Cambridge Biomedical Research Centre, Addenbrooke's Hospital, Cambridge, UK

## Abstract

**Background:**

Obesity is a major risk factor for adverse outcomes after infection with SARS-CoV-2. We aimed to examine this association, including interactions with demographic and behavioural characteristics, type 2 diabetes, and other health conditions.

**Methods:**

In this prospective, community-based, cohort study, we used de-identified patient-level data from the QResearch database of general practices in England, UK. We extracted data for patients aged 20 years and older who were registered at a practice eligible for inclusion in the QResearch database between Jan 24, 2020 (date of the first recorded infection in the UK) and April 30, 2020, and with available data on BMI. Data extracted included demographic, clinical, clinical values linked with Public Health England's database of positive SARS-CoV-2 test results, and death certificates from the Office of National Statistics. Outcomes, as a proxy measure of severe COVID-19, were admission to hospital, admission to an intensive care unit (ICU), and death due to COVID-19. We used Cox proportional hazard models to estimate the risk of severe COVID-19, sequentially adjusting for demographic characteristics, behavioural factors, and comorbidities.

**Findings:**

Among 6 910 695 eligible individuals (mean BMI 26·78 kg/m^2^ [SD 5·59]), 13 503 (0·20%) were admitted to hospital, 1601 (0·02%) to an ICU, and 5479 (0·08%) died after a positive test for SARS-CoV-2. We found J-shaped associations between BMI and admission to hospital due to COVID-19 (adjusted hazard ratio [HR] per kg/m^2^ from the nadir at BMI of 23 kg/m^2^ of 1·05 [95% CI 1·05–1·05]) and death (1·04 [1·04–1·05]), and a linear association across the whole BMI range with ICU admission (1·10 [1·09–1·10]). We found a significant interaction between BMI and age and ethnicity, with higher HR per kg/m^2^ above BMI 23 kg/m^2^ for younger people (adjusted HR per kg/m^2^ above BMI 23 kg/m^2^ for hospital admission 1·09 [95% CI 1·08–1·10] in 20–39 years age group *vs* 80–100 years group 1·01 [1·00–1·02]) and Black people than White people (1·07 [1·06–1·08] *vs* 1·04 [1·04–1·05]). The risk of admission to hospital and ICU due to COVID-19 associated with unit increase in BMI was slightly lower in people with type 2 diabetes, hypertension, and cardiovascular disease than in those without these morbidities.

**Interpretation:**

At a BMI of more than 23 kg/m^2^, we found a linear increase in risk of severe COVID-19 leading to admission to hospital and death, and a linear increase in admission to an ICU across the whole BMI range, which is not attributable to excess risks of related diseases. The relative risk due to increasing BMI is particularly notable people younger than 40 years and of Black ethnicity.

**Funding:**

NIHR Oxford Biomedical Research Centre.

## Introduction

Early in the COVID-19 pandemic, obesity was implicated as a clinically significant risk factor for severe disease.^1^, ^2^ Multiple studies have supported this theory, and several systematic reviews and meta-analyses on this subject have been published to date.^3^, ^4^, ^5^ However, this association might occur due to a special form of collider bias, termed index event bias.^6^ Almost all studies of this potential association to date have examined outcomes of patients admitted to hospital and compared progression to intensive care unit (ICU) or death between those with and without obesity. Because either obesity itself or the severity of COVID-19 disease could prompt admission to hospital, the association between these factors might be spurious. A large population-based study, which avoided the risk of collider bias, found that having a body-mass index (BMI) of 30 kg/m^2^ or higher was associated with a slightly greater risk of death from COVID-19 than a BMI of less than 30 kg/m^2^.^7^ However, this study did not examine the risk of unit increases in BMI across the population, of which a large proportion have a BMI lower than 30 kg/m^2^.

Some studies have found that male gender, some ethnic groups, and people with type 2 diabetes and other chronic conditions might be at higher risk of adverse outcomes from severe SARS-CoV-2 infection.^7^, ^8^, ^9^, ^10^, ^11^ Whether these characteristics interact with the effect of excess weight is unclear.

Here we report results of a large, representative community-based cohort study of 6·9 million people in England, UK, to thoroughly characterise the association between BMI and severe COVID-19 outcomes and to explore interactions with demographic characteristics and other known risk factors.

**Research in context**

**Evidence before this study**

Several systematic reviews and meta-analyses have reported that obesity is associated with adverse outcomes after infection with SARS-CoV-2 but most studies included people admitted to hospital with COVID-19 symptoms and so could be potentially affected by collider bias, where obesity itself and the severity of COVID-19 disease increase the likelihood of hospital admission. A previous population-based study focused on the effect of obesity (BMI ≥30 kg/m^2^) but did not take into account the consequences of excess weight (ie, a BMI of >25 kg/m^2^) for the majority of the population who do not have obesity. Whether excess weight poses the same risk for all demographic groups or people with chronic health conditions is unclear.

**Added value of this study**

In this very large, community-based cohort study, we found that the hazard ratio of severe outcomes from COVID-19 (ie, admission to hospital, admission to ICU, or death) increase progressively above a BMI of 23 kg/m^2^, which is not attributable to excess risks of related diseases such as type 2 diabetes. We found that BMI is a greater risk factor for younger people (aged 20–39 years) than for older people (≥80 years), and for Black people than for White people.

**Implications of all the available evidence**

Even a small increase in BMI above 23 kg/m^2^ is a risk factor for adverse outcomes after infection with SARS-CoV-2. People with excess weight, even without other comorbidities, are at substantially increased risk of admission to hospital and ICU and death due to COVID-19, especially for younger adults and Black people. Excess weight is a modifiable risk factor and investment in the treatment of overweight and obesity and long-term preventive strategies could help reduce the severity of COVID-19 disease.

## Methods

### Study design and participants

In this prospective, community-based, cohort study, we used data from an anonymised research database of patients from over 1500 English general practices (QResearch version 44). The data include demographic information, medical diagnoses, prescriptions, referrals, laboratory results, and clinical values linked with data from Public Health England's (PHE's) database of SARS-CoV-2 positive PCR tests, and to Hospital Episode Statistics and death certificates from the Office for National Statistics.

We extracted deidentified patient-level data between Jan 24, 2020 (date of the first recorded infection in the UK), and April 30, 2020, from the QResearch database for all individuals aged 20–99 years who were registered at a general practice (GP) that contributes to the QResearch database and had available BMI data.

The QResearch database was approved by the East Midlands Derby Research Ethics Committee (reference 18/EM/0400), which waived the need for written informed consent for collection of deidentified patient data. In accordance with this ethical approval, the protocol for this study was reviewed and approved by the QResearch Scientific Advisory Committee, before they provided approval for our access to the data for the purposes of this project (study reference Q85)

### Procedures

We followed-up the eligible cohort until the earliest occurrence of an outcome of interest, death from other causes, transfer to another practice, or the end of the study period (April 30, 2020).

The main exposure was BMI, calculated as weight in kg divided by height in m squared (kg/m^2^). Weight and height were taken from the GP medical records, either from self-reported measurements or measured at the practice with clothes on. From all available BMI measurements, the last measure of BMI before study entry (recorded before January, 2020) was used and entered in the models as a continuous variable.

For the association of BMI with severe COVID-19 outcomes, we analysed the following confounders: age; GP-recorded sex; self-reported ethnicity (classified as White, Asian, Black, Chinese, and other ethnic group); socioeconomic status (classified into quintiles using the Townsend score,^12^ which is comparable with the index of multiple deprivation^13^ calculated for individual participants); geographical region; smoking status (divided into never smoking, ex-smoker, or light [1–9 cigarettes per day], moderate [10–19 cigarettes per day], or heavy smoker [≥20 cigarettes per day]); non-obesity-related morbidity, including conditions related to severe COVID-19 disease (namely, chronic obstructive pulmonary disease, asthma, autoimmune diseases [systemic lupus erythematosus, rheumatoid diseases], ulcerative colitis or Crohn's disease, type 1 diabetes, chronic liver disease, chronic renal disease, chronic neurological disease, and cerebral palsy); obesity-related morbidity, including hypertension, cardiovascular disease (including congestive heart failure and stroke), reflux disease or gastro-oesophageal reflux disease, and sleep apnoea; and type 2 diabetes. Missing data on any of these confounders were coded as “unknown” and entered as a separate category.

### Outcomes

Outcomes of interest, which were proxy measures of severe COVID-19 outcomes, were: admission to hospital, defined as an having an International Classification of Diseases 10th edition (ICD-10) code in their hospital record for either confirmed (U07.1) or suspected COVID-19 (U07.2) as primary or secondary cause, or new admission to hospital and a positive SARS-CoV-2 PCR test recorded in the PHE linked dataset within 30 days of admission; admission to the ICU, defined as testing positive for SARS-CoV-2 from the PHE records and identified from Hospital Episode Statistics records as being admitted to the ICU with no time limit on when they were admitted; and death, defined using ICD-10 codes on Office for National Statistics death certificates for confirmed or suspected death due to COVID-19 (primary or secondary cause).

### Statistical analysis

We followed a prespecified statistical analysis plan, with the only deviation to exclude the analysis of markers of ectopic fat, for which we did not have access to accurate proxy measures. We used multivariable Cox proportional hazard models with follow-up time in days as the timescale variable to obtain hazard ratios (HRs) with 95% CIs per unit increase in BMI; model 1 [unadjusted]), with sequential adjustment for sex and age (model 2), demographic factors (model 3), smoking (model 4), non-obesity-related morbidity (model 5), and obesity-related morbidity (model 6; fully adjusted). We also calculated the independent associations between the outcomes and with type 2 diabetes, the disease most strongly associated with BMI, and we generated a subsequent model (model 7) that included BMI and type 2 diabetes to mutually adjust them. We assessed the proportional hazards assumption using Schoenfeld residuals and the assumption was not violated. We generated restricted cubic splines models with the same covariate specification as the fully adjusted model with five knots to examine non-linear associations between BMI and the different outcomes.^14^ We prespecified the use of five knots to provide enough flexibility to the model while also not making the model too oversensitive to the smallest fluctuations.^15^, ^16^ After visually inspecting non-linear associations from spline models, we categorised BMI into deciles to determine the nadir and subsequently restricted the main Cox models already described to individuals with a BMI of 23 kg/m^2^ or higher, from which linear and positive associations were identified, for hospital admission and death outcomes.

We fitted multiplicative interaction terms in model 6 to examine heterogeneity in the associations of BMI with severe COVID-19 outcomes by age, sex, self-reported ethnicity, and presence of type 2 diabetes, hypertension, cardiovascular disease, sleep apnoea, and gastro-oesophageal reflux disease. We calculated likelihood ratios to determine p values for heterogeneity without correction for multiple testing. Additionally, we calculated cumulative incidences, and attributable risks and attributable fractions by age group and by BMI, because of the very large differences in absolute risk between age groups.

BMI is not frequently updated or recorded in medical records, particularly for those with no special reason to do so. As such, any associations identified might be biased by measurements of BMI taken many years before exposure to SARS-CoV-2 and hence cause non-differential misclassification. Hence, we did a sensitivity analysis in which we confined the analysis to individuals with BMI recorded within a year of study entry and we used the same procedures as described here to determine the nadir in this group, which was found to be a BMI of 25 kg/m^2^ for hospital admission and a BMI of 28 kg/m^2^ for death. ICU admission data are presented across the full BMI range. We did a second sensitivity analysis in which we excluded people living in care homes, to reduce the possibility of reverse causality, and used the same BMI nadirs as in the main analyses. Furthermore, a proportion of participants had no recorded BMI but the likelihood of having a recorded BMI might be related to the presence of comorbid conditions, especially those related to obesity. We examined this potential bias by comparing demographic characteristics and outcome prevalence between people with and without BMI recorded; and examining the difference in risk of severe COVID-19 outcomes between people with and without BMI recorded. For all sensitivity analyses we used fully adjusted Cox proportional hazards model (model 6).

We did all analyses using Stata (version 16) and two-tailed p values of less than 0·01 were considered to be statistically significant.

### Role of the funding source

The funder of the study had no role in the study design, data collection, data interpretation, data analysis, or writing of the report.

## Results

Between Jan 24 and April 30, 2020, data were available for 10 594 500 patients from 2205 GP practices. 8 139 662 individuals were aged 20–99 years, of whom 6 910 695 (84·9%) had at least one BMI measurement (mean 26·78 kg/m^2^ [SD 5·59]) and were included in the main analyses (table 1; appendix p 2). The cohort was followed-up until April 30, 2020. During the study period, 13 503 hospital admissions due to COVID-19, 1601 ICU admissions due to COVID-19, and 5479 deaths due to COVID-19 occurred. Approximately a third of those with severe COVID-19 outcomes as indicated by being admitted to hospital, ICU, or those who died of COVID-19 were classified as having type 2 diabetes (table 1). Most people with severe COVID-19 were aged 60 years or older.

**Table 1:** Summary of baseline characteristics of the study population

		**Total population (n=6 910 695)**	**Hospitalised with COVID-19 (n=13 503)**	**Admitted to ICU with COVID-19 (n=1601)**	**Death from COVID-19 (n=5479)**
BMI, kg/m^2^		26·78 (5·59)	28·62 (6·23)	30·56 (6·17)	27·25 (6·18)
Type 2 diabetes		577 246 (8·4%)	4256 (31·5%)	537 (33·5%)	1919 (35·0%)
BMI categories, kg/m^2^					
	<18·5	209 497 (3·0%)	362 (2·7%)	12 (0·7%)	275 (5·0%)
	18·5 to <25	2 713 189 (39·3%)	3655 (27·1%)	289 (18·1%)	1871 (34·1%)
	25 to <30	2 306 897 (33·4%)	4593 (34·0%)	513 (32·0%)	1751 (32·0%)
	30 to <35	1 072 777 (15·5%)	2823 (20·9%)	421 (26·3%)	964 (17·6%)
	≥35	608 335 (8·8%)	2070 (15·3%)	366 (22·9%)	618 (11·3%)
Age group, years					
	20 to 39	2 384 223 (34·5%)	922 (6·8%)	127 (7·9%)	32 (0·6%)
	40 to 59	2 444 011 (35·4%)	2845 (21·1%)	582 (36·4%)	378 (6·9%)
	60 to 79	1 652 615 (23·9%)	5058 (37·5%)	788 (49·2%)	1928 (35·2%)
	≥80	429 846 (6·2%)	4678 (34·6%)	104 (6·5%)	3141 (57·3%)
Sex					
	Female	3 668 308 (53·1%)	5993 (44·4%)	486 (30·4%)	2304 (42·1%)
	Male	3 242 387 (46·9%)	7510 (55·6%)	1115 (69·6%)	3175 (57·9%)
Self-reported*					
	White	4 771 657 (69·0%)	8780 (65·0%)	869 (54·3%)	3796 (69·3%)
	Asian	550 618 (8·0%)	1355 (10·0%)	264 (16·5%)	413 (7·5%)
	Black	254 507 (3·7%)	1081 (8·0%)	170 (10·6%)	364 (6·6%)
	Chinese	64 984 (0·9%)	62 (0·5%)	11 (0·7%)	24 (0·4%)
	Others or not recorded	1 268 929 (18·4%)	2225 (16·5%)	287 (17·9%)	882 (16·1%)
Townsend deprivation scores					
	Quintile 1 (most affluent)	1 617 387 (23·4%)	2592 (19·2%)	279 (17·4%)	1134 (20·7%)
	Quintile 2	1 536 385 (22·2%)	2640 (19·6%)	282 (17·6%)	1068 (19·5%)
	Quintile 3	1 353 591 (19·6%)	2736 (20·3%)	322 (20·1%)	1218 (22·2%)
	Quintile 4	1 224 177 (17·7%)	2643 (19·6%)	307 (19·2%)	1004 (18·3%)
	Quintile 5 (most deprived)	1 149 438 (16·6%)	2864 (21·2%)	408 (25·5%)	1044 (19·1%)
	Missing	29 717 (0·4%)	28 (0·2%)	<5 (0·2%)†	11 (0·2%)
Region in England					
	East Midlands	182 115 (2·6%)	197 (1·5%)	16 (1·0%)	83 (1·5%)
	East of England	254 006 (3·7%)	452 (3·3%)	55 (3·4%)	190 (3·5%)
	London	1 746 979 (25·3%)	4699 (34·8%)	704 (44·0%)	1646 (30·0%)
	North East	169 713 (2·5%)	303 (2·2%)	33 (2·1%)	105 (1·9%)
	North West	1 237 041 (17·9%)	2610 (19·3%)	257 (16·1%)	1189 (21·7%)
	South Central	928 765 (13·4%)	1579 (11·7%)	169 (10·6%)	723 (13·2%)
	South East	775 900 (11·2%)	1178 (8·7%)	147 (9·2%)	520 (9·5%)
	South West	698 240 (10·1%)	715 (5·3%)	71 (4·4%)	276 (5·0%)
	West Midlands	667 916 (9·7%)	1417 (10·5%)	115 (7·2%)	601 (11·0%)
	Yorkshire & Humber	250 020 (3·6%)	353 (2·6%)	34 (2·1%)	146 (2·7%)
Smoking					
	Never smoking	4 071 381 (58·9%)	7634 (56·5%)	926 (57·8%)	2873 (52·4%)
	Ex-smoker	1 610 196 (23·3%)	4793 (35·5%)	562 (35·1%)	2224 (40·6%)
	Light smoker	90 4031 (13·1%)	823 (6·1%)	85 (5·3%)	275 (5·0%)
	Moderate smoker	185 743 (2·7%)	121 (0·9%)	10 (0·6%)	40 (0·7%)
	Heavy smoker	91 751 (1·3%)	98 (0·7%)	14 (0·9%)	37 (0·7%)
	Missing	47 593 (0·7%)	34 (0·3%)	<5 (0·2%) †	30 (0·5%)
Non-obesity-related morbidity					
	Asthma or chronic obstructive pulmonary disease	1 111 817 (16·1%)	3241 (24·0%)	322 (20·1%)	1291 (23·6%)
	Autoimmune disease	78 479 (1·1%)	402 (3·0%)	43 (2·7%)	158 (2·9%)
	Ulcerative colitis, Crohn's disease	69 773 (1·0%)	211 (1·6%)	24 (1·5%)	77 (1·4%)
	Type 1 diabetes	44 248 (0·6%)	268 (2·0%)	35 (2·2%)	83 (1·5%)
	Chronic liver disease	44 666 (0·6%)	274 (2·0%)	26 (1·6%)	95 (1·7%)
	Chronic renal disease	327 163 (4·7%)	3397 (25·2%)	222 (13·9%)	1863 (34·0%)
	Chronic neurological disease	232 318 (3·4%)	2401 (17·8%)	77 (4·8%)	1981 (36·2%)
	Cerebral palsy	6678 (0·1%)	31 (0·2%)	<5 (0·1%)†	9 (0·2%)
Obesity-related morbidity					
	Hypertension	1 362 614 (19·7%)	7115 (52·7%)	754 (47·1%)	3427 (62·5%)
	Cardiovascular disease	415 794 (6·0%)	3547 (26·3%)	232 (14·5%)	2026 (37·0%)
	Sleep apnoea	91 759 (1·3%)	497 (3·7%)	100 (6·2%)	180 (3·3%)
	Gastro-oesophageal reflux disease	368 489 (5·3%)	1168 (8·6%)	114 (7·1%)	457 (8·3%)
	Congestive heart failure	93 691 (1·4%)	1336 (9·9%)	69 (4·3%)	755 (13·8%)
	Stroke	170 194 (2·5%)	1732 (12·8%)	61 (3·8%)	1065 (19·4%)

**Da**ta are mean (SD) or n (%). Townsend scores were obtained for each patient as a proxy for socioeconomic status.

* Asian includes Indian, Pakistani, Bangladeshi, other Asian; and Black includes Caribbean, and Black African.

† QResearch terms and conditions specify that reports, papers, or statistical tables that are published or released will not identify individual cases or medical practices or enable individual cases or practices to be identified; therefore, data for cells including fewer than five cases cannot be presented here. ICU=intensive care unit.

**Figure 1:**
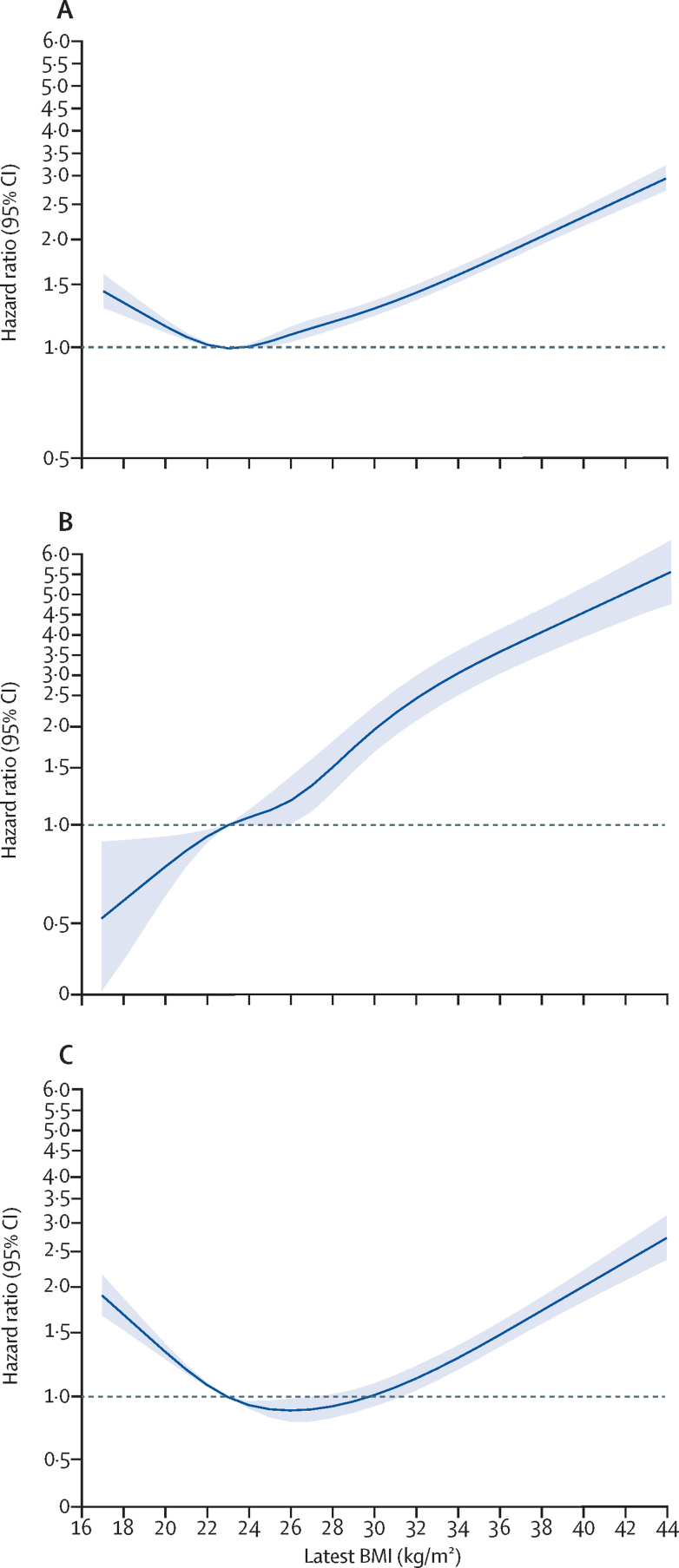
Associations between BMI and COVID-19-related admission to hospital (A), COVID-19-related admission to ICU (B), and death due to COVID-19 (C) in the total population with a BMI measurement (n=6 910 695) Solid line is hazard ratio estimate, with shaded areas showing 95% CIs. The y axis is on a logarithmic scale. Reference BMI of 23 kg/m^2^ was used. ICU=intensive care unit.

We found non-linear associations between BMI and hospital admission and death due to COVID-19, and a linear association between BMI and ICU admission due to COVID-19 (figure 1, table 2). Each excess BMI unit above a BMI of 23 kg/m^2^ was associated with increased risk of hospital admission (adjusted HR 1·05 [95% CI 1·05–1·05]), ICU admission (1·10 [1·09–1·10]), and death (1·04 [1·04–1·05]) in the fully adjusted model (model 6). The spline models showed an increased HR for hospital admission and death due to COVID-19 among people with a BMI of less than 23 kg/m^2^, although the association with ICU admission remained linear across the entire BMI range (figure 1).

**Table 2:** Associations between BMI and type 2 diabetes with severe COVID-19

	**BMI**	**Type 2 diabetes**
Hospital admission*		
Model 1 (unadjusted)	1·05 (1·05–1·06)	5·09 (4·91–5·28)
Model 2 (model 1 plus age and sex)	1·06 (1·06–1·07)	2·46 (2·37–2·55)
Model 3 (model 2 plus demographic factors)	1·06 (1·06–1·07)	2·08 (2·00–2·16)
Model 4 (model 3 plus smoking)	1·06 (1·06–1·06)	2·06 (1·98–2·14)
Model 5 (model 4 plus non-obesity-related morbidity)	1·06 (1·05–1·06)	1·86 (1·78–1·93)
Model 6 (model 5 plus obesity-related morbidity)	1·05 (1·05–1·05)	1·72 (1·65–1·79)
Model 7 (model 6 plus BMI and type 2 diabetes)†	1·04 (1·04–1·05)	1·59 (1·52–1·66)
**ICU admission**		
Model 1 (unadjusted)	1·10 (1·09–1·11)	5·58 (5·03–6·19)
Model 2 (model 1 plus age and sex)	1·10 (1·10–1·11)	3·58 (3·21–4·00)
Model 3 (model 2 plus demographic factors)	1·11 (1·10–1·12)	2·88 (2·57–3·22)
Model 4 (model 3 plus smoking)	1·11 (1·10–1·12)	2·82 (2·52–3·16)
Model 5 (model 4 plus non-obesity-related morbidity)	1·11 (1·10–1·11)	2·66 (2·37–2·99)
Model 6 (model 5 plus obesity-related morbidity)	1·10 (1·09–1·10)	2·32 (2·05–2·61)
Model 7 (model 6 plus BMI and type 2 diabetes)†	1·09 (1·08–1·10)	1·89 (1·67–2·13)
Death*		
Model 1 (unadjusted)	1·03 (1·02–1·03)	5·96 (5·64–6·30)
Model 2 (model 1 plus age and sex)	1·06 (1·05–1·06)	2·23 (2·11–2·36)
Model 3 (model 2 plus demographic factors)	1·05 (1·05–1·06)	1·92 (1·81–2·03)
Model 4 (model 3 plus smoking)	1·05 (1·04–1·06)	1·90 (1·79–2·01)
Model 5 (model 4 plus non-obesity-related morbidity)	1·05 (1·04–1·06)	1·76 (1·66–1·87)
Model 6 (model 5 plus obesity-related morbidity)	1·04 (1·04–1·05)	1·66 (1·56–1·76)
Model 7 (model 6 plus BMI and type 2 diabetes)†	1·04 (1·04–1·05)	1·64 (1·54–1·75)

Data are hazard ratios with 95% CI in parentheses. Hazard ratios are per unit increase in BMI.

* Associations with BMI and hospital admission and death were restricted to individuals with a BMI of ≥23 kg/m^2^

† Model 7 is mutually adjusted for BMI and type 2 diabetes. ICU=intensive care unit.

The associations of BMI with severe COVID-19 outcomes remained significant although slightly attenuated after adjusting for the presence of type 2 diabetes in model 7 (table 2). We also found significant associations between presence of type 2 diabetes and severe COVID-19 outcomes, even after mutual adjustment for BMI in model 7 (table 2).

We did a sensitivity analyses including only individuals with a BMI measurement in the past year (between Jan 23, 2019, and Jan 23, 2020). 2 291 940 participants had a BMI measurement in this timeframe, among whom we found that each unit increase in BMI was associated with increased risk of hospital admission due to COVID-19 (HR per unit BMI increase of 1·05 [95% CI 1·04–1·05]) among individuals with a BMI of 25 kg/m^2^ or higher, and with ICU admission (1·08 [1·07–1·10]) across the entire BMI range, and death due to COVID-19 (1·06 [1·04–1·07]) among individuals with a BMI of 28 kg/m^2^ or higher in fully adjusted model (model 6; appendix pp 6–7). A second sensitivity analysis, excluding people living in care homes, showed increased risk of hospital admission due to COVID-19 (HR per unit increase in BMI of 1·05 [1·05–1·06]) in individuals with a BMI of 23 kg/m^2^ or higher, of ICU admission (1·10 [1·09–1·11]) across the entire BMI range, and of death (1·06 [1·05–1·06]) in individuals with a BMI of 23 kg/m^2^ or higher (appendix p 7). A third sensitivity analysis excluding participants with missing data on important confounders also showed consistent results with the main analysis (appendix p 7).

**Figure 2:**
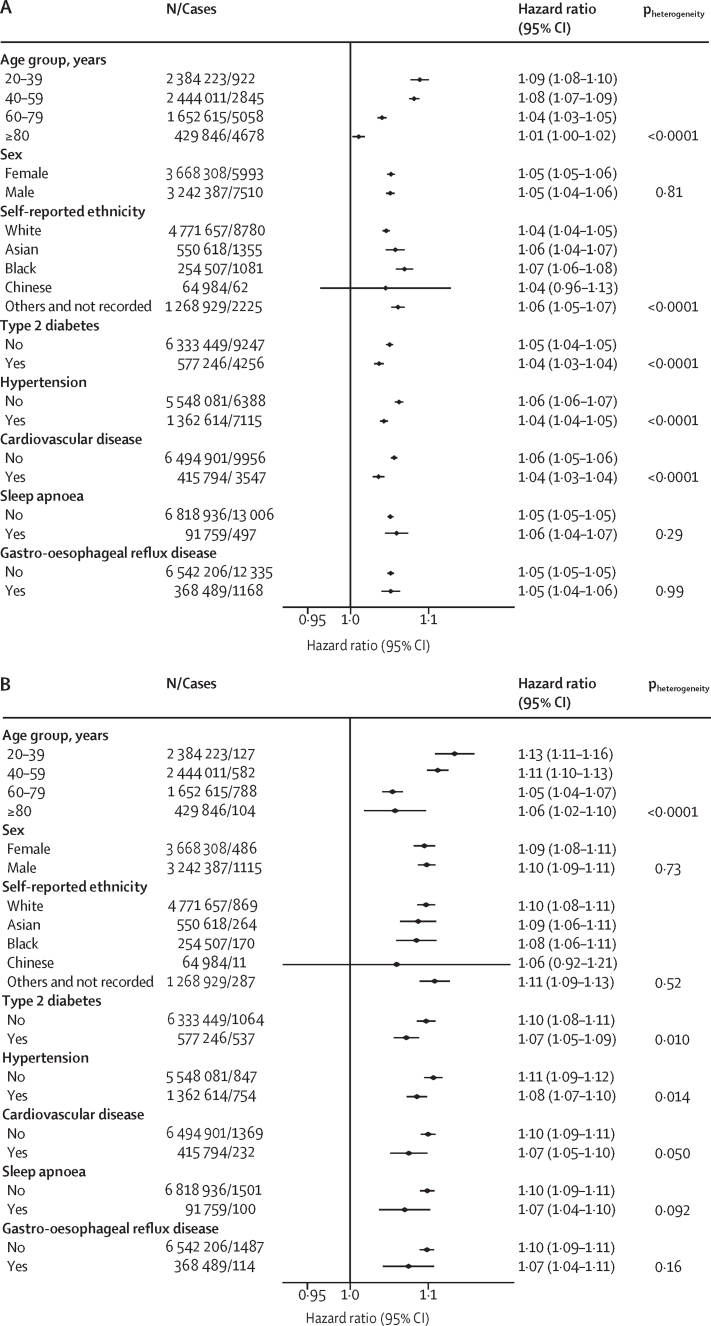

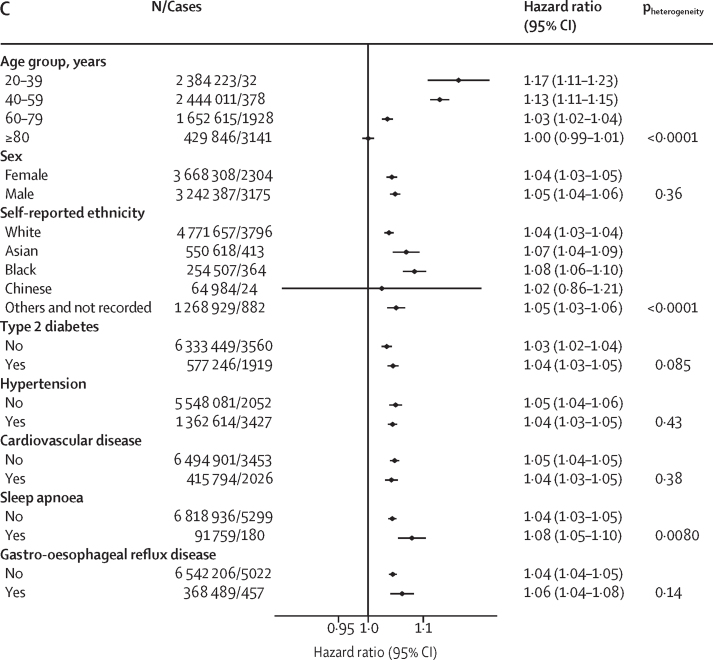
Associations between BMI and COVID-19-related admission to hospital (A), COVID-19-related admission to ICU (B), and death due to COVID-19 (C) in the total population with a BMI measurement (n=6 910 695), by age group, sex, ethnicity, and morbidity Hazard ratios are per BMI unit increase, and were calculated using the fully adjusted model (model 6) and likelihood ratio tests were calculated to determine p_heterogeneity_ without correction for multiple testing. Associations reported in this figure are restricted to those with a BMI of ≥23 kg/m^2^ for hospital admission and death. ICU=intensive care unit.

Age modified the association between BMI and severe COVID-19 outcomes significantly (p<0·0001 for all outcomes; figure 2). The HR was highest in the youngest age groups and decreased progressively with increasing age becoming non-significant in the 80 years and older age group for death. Among people aged 20–39 years, each BMI unit increase above 23 kg/m^2^ was associated with increased risk of hospital admission (HR 1·09 [95% CI 1·08–1·10]), ICU admission (1·13 [1·11–1·16]), and death due to COVID-19 (1·17 [1·11–1·23; figure 2). The incidence of severe COVID-19 increased with age, therefore the attributable risks were generally higher in people aged 40–59 and 60–69 years but attributable fractions were higher in those aged 20–39 years (appendix p 7). We found a significant interaction between BMI and self-reported ethnicity (p<0·0001) for hospital admission and death due to COVID-19, with Black people having a higher risk than White people of hospital admission due to COVID-19 (1·07 [1·06–1·08] *vs* 1·04 [1·04–1·05]) and of death due to COVID-19 (1·08 [1·06–1·10] *vs* 1·04 [1·03–1·04]), but not ICU admission (figure 2). We found no differences between other ethnic groups and those with self-reported White ethnicity for these outcomes. We found no difference in effect estimates by sex for any of the assessed outcomes (figure 2).

We found that increasing BMI was associated with slightly greater HR for admission to hospital and admission to ICU for people without type 2 diabetes than those with type 2 diabetes, those with hypertension than those without hypertension, and those with cardiovascular disease than those without cardiovascular disease (figure 2). We found no significant interaction between these morbidities and risk of death due to COVID-19, except for in people with sleep apnoea in whom a greater HR for death due to COVID-19 was seen per extra BMI unit increase than in those without sleep apnoea (p=0·0080).

Among the 1 228 967 people without a recorded BMI, a higher proportion were younger than 40 years (825 116 [67·1%] aged 20–39 years) and a smaller proportion had type 2 diabetes (11 700 [1·0%]) than among those with available BMI measurements (appendix p 8) Additionally, among those without a recorded BMI measurement, a higher proportion of people had missing self-reported ethnicity (573 669 [46·7%]) and smoking status (288 780 [23·5%]) than among those with available BMI measurements. We found no difference in the risk of admission to hospital due to COVID-19 between those with and without a BMI measurement (appendix p 9). We found that those without a BMI measurement were less likely to be admitted to an ICU due to COVID-19 (HR 0·70 [95% CI 0·56–0·88]) and were more likely to die from COVID-19 (1·38 [1·20–1·59]) than those with a BMI measurement (appendix p 9).

## Discussion

We found a significant positive linear association between increasing BMI and admission to ICU due to COVID-19, with significantly higher risk for every BMI unit increase. We found J-shaped associations between increasing BMI and hospital admission or death due to COVID-19, with increased risks for people with a BMI of 20 kg/m^2^ or less and approximately linear increases in risk for people with a BMI of more than 23 kg/m^2^ for admission to hospital, but the risk of death increased only in people with a BMI of more than 28 kg/m^2^. These outcomes were largely independent of other health conditions, including type 2 diabetes.

A previous study reported that the risk of COVID-19-related death was more strongly associated with BMI for people aged 70 years or younger, compared with older people.^17^ Our findings support this observation, with risk of COVID-19-related death per BMI unit increase being highest in the youngest age groups, decreasing with increasing age. Nevertheless, the incidence of severe COVID-19 outcomes in young adults was low, which meant the attributable risk was greatest in people of middle age (40–59 years). Our large sample also allowed us to assess other potential interactions. We found no evidence of an interaction between BMI and sex or Asian and Chinese ethnicity, although the risks associated with higher BMI were amplified for people of Black ethnicity compared with those of White ethnicity.

A previous population-based cohort study reported that the risks of COVID-19-related death associated with obesity were lower for people with high blood pressure or a diagnosis of hypertension than the rest of the population.^7^ We did not find this association to be true after extensive adjustment for confounders, but we did observe that the risks of admission to hospital and ICU due to COVID-19 associated with unit increase in BMI were lower for people with type 2 diabetes, hypertension, and cardiovascular disease than among those without each of these morbidities. In a previous study, people on thiazide diuretics, calcium antagonists, angiotensin-converting enzyme inhibitors, and angiotensin receptor blockers had lower risk of severe COVID-19 than people not taking these drugs.^18^ However, whether these drugs interact with obesity such that they selectively reduce the risk of severe COVID-19 apparently conferred by obesity is unknown and this association could be a result of confounding.

In our study, the association between BMI and hospital admission and death due to COVID-19 was J-shaped, presumably because both of these events are increased by frailty,^19^ which is commonly associated with low BMI.^20^ By contrast, the association between BMI and admission to ICU was linear across the entire BMI range. ICU admission is unlikely in people with pre-existing frailty, so this observation might indicate an underlying biological association between increasing weight and risk of severe COVID-19.

Male sex, Black and Asian ethnicity, and type 2 diabetes have been found to be associated with an increased risk of adverse outcomes from severe SARS-CoV-2 infection.^7^, ^8^, ^9^, ^10^, ^11^ One factor that links these groups is a tendency to store fat in the abdominal region (visceral fat) and also in tissues other than adipose tissue, such as the liver, heart, or skeletal muscle (ie, ectopic fat).^21^ Three small studies of patients admitted to hospital due to COVID-19 have found that a tendency for visceral fat accumulation, measured using CT, was independently associated with adverse COVID-19-related outcomes.^22^, ^23^, ^24^ The risk of severe COVID-19 outcomes attributable to excess weight (ie, ≥23 kg/m^2^) has been proposed to be a consequence of metabolic impairment of organ functioning, leading to insulin resistance.^25^ We found little evidence of a difference in the association between BMI and severe COVID-19 outcomes in people with and without type 2 diabetes (a condition strongly associated with accumulation of ectopic fat) to support this hypothesis. In fact, we found that people with type 2 diabetes were at lower risk of severe COVID-19 outcomes per unit increase in BMI than those without type 2 diabetes. People with type 2 diabetes are only a subset of people with increased likelihood of ectopic fat deposition. Central fat accumulation could contribute to the increased risk associated with unit increase in BMI in the younger age group, since previous reports suggest a disproportionate increase in waist circumference relative to BMI among English adults.^26^ Direct measurements of ectopic fat, or proxy measures such as waist circumference, are rarely done in routine care and accurate data were not available for our analysis so we were unable to test this hypothesis, as was originally specified in our protocol.

Another possible explanation for the increased risks associated with excess weight among people who become seriously ill with COVID-19 include difficulties in intubation and difficulties in positioning and movement by nursing staff, which might complicate recovery times.^27^ However, a study of seasonal influenza infection in the USA did not find any evidence that obesity (BMI ≥30 kg/m^2^) was a risk factor for requiring mechanical ventilation or death,^28^ and a meta-analysis of the effect of excess weight on clinical outcomes in five studies including over 6000 patients reported that obesity (also defined as BMI ≥30 kg/m^2^) was associated with decreased mortality in patients admitted to the ICU with adult respiratory distress syndrome,^29^ a condition that is symptomatic of COVID-19.

The mechanisms underpinning the association between obesity and severe COVID-19 outcomes remain elusive. The risk associated with obesity has been suggested to be due to a pro-inflammatory state that seems to be crucial in causing other weight-related diseases, but our adjustment for these conditions, which are on the causal pathway, did not abolish most of the excess risk. However, since most other obesity-related risks are improved with weight loss, weight-loss interventions might reduce COVID-19 disease severity. Although we originally planned to investigate this hypothesis in our protocol, we were unable to because the number of participants reported to have been offered referrals to weight management programmes was low and weight change was poorly recorded.

The strengths of this study include the large sample size and representative population. We were able to study associations with COVID-19-related outcomes across the full BMI range, and found a linear increase in risk at a BMI of more than 23 kg/m^2^. We also found increased risk of severe COVID-19 outcomes at low BMI, even within the so-called healthy range (19–25 kg/m^2^), for admission to hospital and death due to COVID-19. Limitations of our study include that the analysis of the effect of BMI might be limited by the small sample of people with more recent BMI measurements (ie, within the past year). In a sensitivity analysis that excluded individuals with measures of BMI recorded more than a year before cohort entry, we found no substantial change in the associations observed. Daily fluctuations in weight and errors caused by clothing will cause imprecision in estimates of BMI. We did not attempt to correct for these errors, therefore precise definition of the BMI associated with minimum risk might not be possible, although it is unlikely to differ by more than 1 BMI unit from any estimate from these data.

Substantial bias might have been introduced by people with no recording of BMI. Two-thirds of these people were aged 20–39 years and they were much less likely to have comorbidities than people in the cohort with BMI measurements, who were mostly aged 60 years and older. Those without BMI measurements were less likely to be admitted to an ICU due to COVID-19 than people with a recorded BMI, but more likely to die due to COVID-19, which is perhaps indicative that their risk of poor outcomes was undetected. However, a large proportion of people without a BMI measurement were also missing data on ethnicity and smoking status, so residual confounding might affect this association. Another limitation is that, given the multiple testing for interactions, we cannot rule out the possibility of chance findings, but we found consistency in patterns across outcomes. Finally, as with any observational analyses, residual confounding due to unmeasured covariates (eg, population density and occupation) might have occurred.

Our findings from this large population-based cohort emphasise that excess weight is associated with substantially increased risks of severe COVID-19 outcomes, and one of the most important modifiable risk factors identified to date. This risk factor is already included in the QCOVID risk calculator^13^ and, together with the interactions observed with age and Black ethnicity, might be important in determining priorities for vaccination against SARS-CoV-2 infection. Although as yet unproven, interventions that reduce weight might reduce the risk of severe COVID-19 outcomes in individuals. In the longer term, our findings highlight the need to work towards a healthy weight at a population level. Additionally, such efforts towards achieving a healthy weight should help to reduce the risk of type 2 diabetes, cardiovascular disease, and some cancers, which have continued throughout the pandemic and which place ongoing burdens on health-care systems.

## Data sharing

The study protocol and statistical analysis plan for this project are available on request from the corresponding author. De-identified individual participant data that underlie the results reported in this Article will be made available, with requests accepted immediately after publication, for proposals that set out to achieve aims specified in a methodologically and scientifically sound protocol that are approved by the QResearch Scientific Advisory Committee (“learned intermediary”), where costs of providing access to the data are covered, where requests are compliant with the legal permissions of QResearch data providers, and QResearch data security requirements are met. Information regarding submission of applications to access data can be found online.

## Declaration of interests

NMA, PA, and SAJ were investigators on a trial of total diet replacement for weight loss funded by a grant from Cambridge Weight Plan UK to the University of Oxford (Oxford, UK) and received no personal payments from this. PA spoke at a symposium at the Royal College of General Practitioners annual conference on interventions for weight loss that was funded by Novo Nordisk and received no personal payments. JH-C reports grants from National Institute for Health Research (NIHR) Oxford Biomedical Research Centre (BRC), John Fell Oxford University Press Research Fund, Cancer Research UK (C5255/A18085) through the Cancer Research UK Oxford Centre, and Oxford Wellcome Institutional Strategic Support Fund (204826/Z/16/Z) during the conduct of the study. JH-C received personal fees and other support from ClinRisk (until 2019) outside of the submitted work, and is an unpaid director of QResearch, a not-for-profit organisation that is a partnership between the University of Oxford and Egton Medical Information Systems (EMIS) Health who supply the QResearch database used for this work. All other authors declare no competing interests.
