# Supplementary material for: Associations between body-mass index and COVID-19 severity in 6·9 million people in England: a prospective, community-based, cohort study
Source: Lancet Diabetes Endocrinol. 2021 Jun;9(6):350–9. doi: 10.1016/S2213-8587(21)00089-9 (PMC8081400; doi:10.1016/S2213-8587(21)00089-9)
Supplement: Supplementary appendix [file mmc1.pdf]

THE LANCET
Diabetes & Endocrinology

Supplementary appendix

This appendix formed part of the original submission and has been peer reviewed. We post it as supplied by the authors.

Supplement to: Gao M, Piernas C, Astbury N M, et al. Associations between body-mass index and COVID-19 severity in 6·9 million people in England: a prospective, community-based, cohort study. *Lancet Diabetes Endocrinol* 2021; published online April 28. https://doi.org/10.1016/S2213-8587(21)00089-9.

Supplementary Tables and Figures

Supplementary Figure 1. Study flow diagram. 2

Supplementary Table 1. Baseline characteristics of the study population by BMI group..... 3

Supplemental Table 2. Attributable risks and fractions in risk of severe COVID-19 outcomes associated with BMI across age groups.. 5

Supplementary Figure 2. Associations of BMI with severe COVID-19 outcomes: sensitivity analysis using BMI measurements taken over the previous year (n=2,291,940) . 6

Supplemental Table 3. Sensitivity analyses of BMI with severe COVID-19 outcomes excluding people with recorded BMI before 24th January 2019, excluding people living in care homes and excluding people with missing covariates 7

Supplemental Table 4. Baseline characteristics in people with vs without BMI recorded.... 8

Supplemental Table 5. Risk of severe COVID-19 outcomes in people with vs without BMI recorded.. 9

Supplementary Figure 1. Study flow diagram.

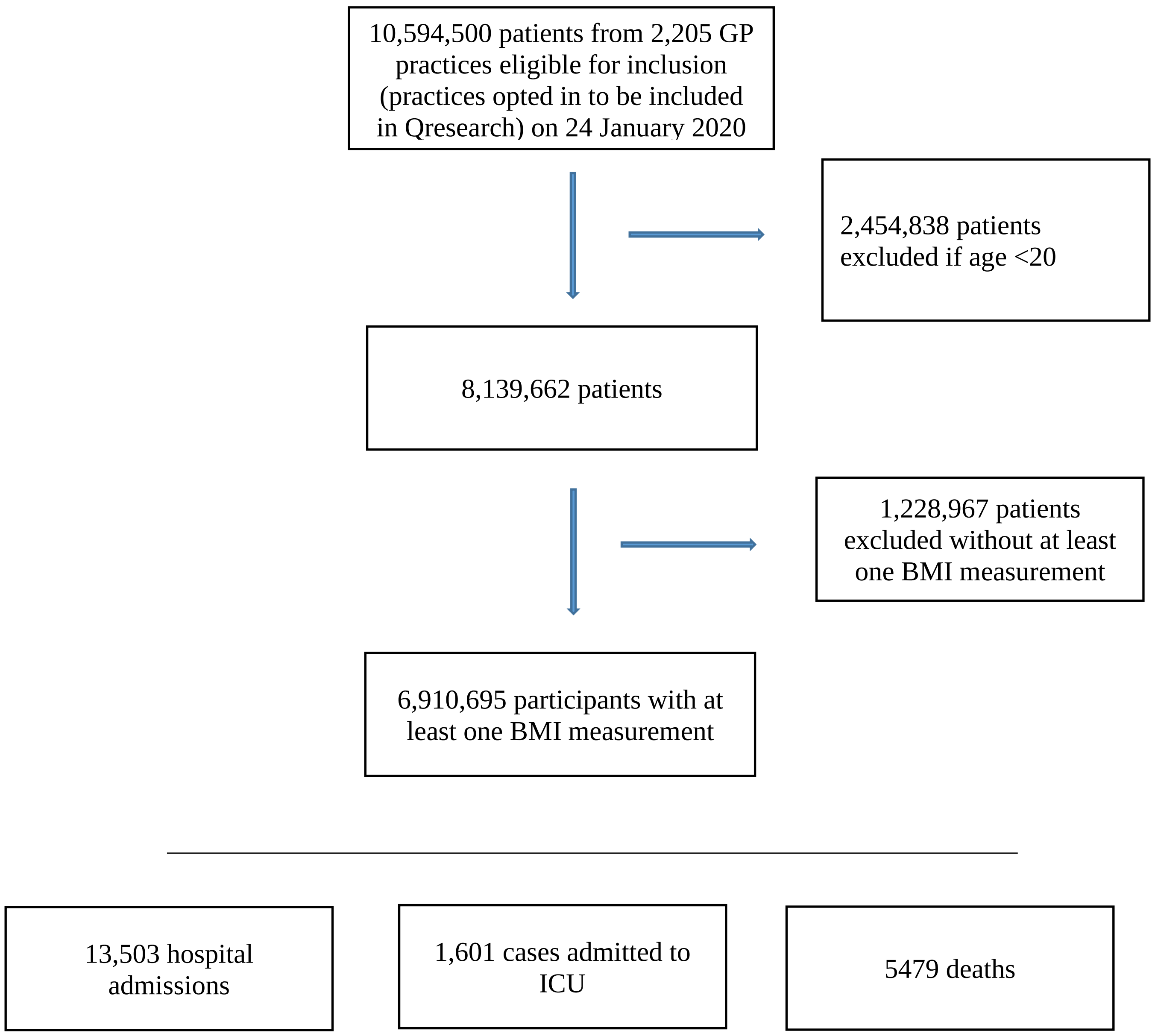

1. Hospital admission: defined as an having ICD-10 code in hospital record for either confirmed (U07.1) or suspected COVID-19 (U07.2) as primary or secondary cause, or new hospital admission associated with confirmed COVID-19 within 30 days of the test;
2. ICU Admission: defined as testing positive for SARS-CoV-2 and identified from ICNARC records;
3. Death: defined using ICD-10 codes on ONS death certificates for confirmed or suspected death from COVID-19 (primary or secondary cause).

Supplementary Table 1. Baseline characteristics of the study population by BMI group

	Underweight (<18.5)	Healthy weight (18.5 to <25)	Overweight (25 to <30)	Obesity I (30-34.9)	Obesity II/III (35+)
**N**	209497	2713189	2306897	1072777	608335
**COVID-19 Death**	275 (0.1%)	1871 (0.1%)	1751 (0.1%)	964 (0.1%)	618 (0.1%)
**COVID-19 hospital admission**	362 (0.2%)	3655 (0.1%)	4593 (0.2%)	2823 (0.3%)	2070 (0.3%)
**COVID-19 ICU admission**	12 (<1%)	289 (<1%)	513 (<1%)	421 (<1%)	366 (0.1%)
**Exposures**					
BMI, mean (SD)	17.31 (0.91)	22.30 (1.72)	27.23 (1.41)	32.06 (1.41)	39.03 (3.27)
Type 2 diabetes	3801 (1.8%)	99801 (3.7%)	195397 (8.5%)	155284 (14.5%)	122963 (20.2%)
**Age group**					
20-39	141986 (67.8%)	1224605 (45.1%)	614919 (26.7%)	245552 (22.9%)	157161 (25.8%)
40-59	31872 (15.2%)	837028 (30.9%)	885324 (38.4%)	432615 (40.3%)	257172 (42.3%)
60-79	21997 (10.5%)	488263 (18.0%)	641918 (27.8%)	328949 (30.7%)	171488 (28.2%)
80+	13642 (6.5%)	163293 (6.0%)	164736 (7.1%)	65661 (6.1%)	22514 (3.7%)
**Gender**					
Men	86418 (41.3%)	1168743 (43.1%)	1246326 (54.0%)	521641 (48.6%)	219259 (36.0%)
**Ethnicity**					
White	124525 (59.4%)	1846548 (68.1%)	1603035 (69.5%)	757697 (70.6%)	439852 (72.3%)
Asian	22408 (10.7%)	225424 (8.3%)	196989 (8.5%)	75465 (7.0%)	30332 (5.0%)
Black	6013 (2.9%)	76338 (2.8%)	89859 (3.9%)	51579 (4.8%)	30718 (5.0%)
Chinese	6039 (2.9%)	43614 (1.6%)	12308 (0.5%)	2315 (0.2%)	708 (0.1%)
Others/Not recorded	50512 (24.1%)	521265 (19.2%)	404706 (17.5%)	185721 (17.3%)	106725 (17.5%)
**Townsend deprivation scores**					
Quintile 1 (most affluent)	36570 (17.5%)	622503 (22.9%)	590534 (25.6%)	249995 (23.3%)	117785 (19.4%)
Quintile 2	38776 (18.5%)	577439 (21.3%)	537823 (23.3%)	248950 (23.2%)	133397 (21.9%)
Quintile 3	41325 (19.7%)	511735 (18.9%)	445519 (19.3%)	220533 (20.6%)	134479 (22.1%)
Quintile 4	43978 (21.0%)	486180 (17.9%)	381285 (16.5%)	190285 (17.7%)	122449 (20.1%)
Quintile 5 (most deprived)	47883 (22.9%)	502361 (18.5%)	342341 (14.8%)	158901 (14.8%)	97952 (16.1%)
Missing	965 (0.5%)	12971 (0.5%)	9395 (0.4%)	4113 (0.4%)	2273 (0.4%)
**English Region**					
East Midlands	8560 (4.1%)	81371 (3.0%)	54455 (2.4%)	24530 (2.3%)	13199 (2.2%)
East of England	7343 (3.5%)	97268 (3.6%)	88219 (3.8%)	39851 (3.7%)	21325 (3.5%)
London	57656 (27.5%)	766163 (28.2%)	561607 (24.3%)	237393 (22.1%)	124160 (20.4%)
North East	5567 (2.7%)	61099 (2.3%)	54449 (2.4%)	29564 (2.8%)	19034 (3.1%)
North West	35632 (17.0%)	438953 (16.2%)	421946 (18.3%)	213973 (19.9%)	126537 (20.8%)
South Central	25222 (12.0%)	359033 (13.2%)	319845 (13.9%)	143306 (13.4%)	81359 (13.4%)
South East	21572 (10.3%)	299497 (11.0%)	263525 (11.4%)	122151 (11.4%)	69155 (11.4%)
South West	20831 (9.9%)	280775 (10.3%)	228283 (9.9%)	106213 (9.9%)	62138 (10.2%)
West Midlands	18406 (8.8%)	235418 (8.7%)	232175 (10.1%)	114761 (10.7%)	67156 (11.0%)
Yorkshire & Humber	8708 (4.2%)	93612 (3.5%)	82393 (3.6%)	41035 (3.8%)	24272 (4.0%)
**Smoking**					
Non smoker	123806 (59.1%)	1659427 (61.2%)	1340494 (58.1%)	604069 (56.3%)	343585 (56.5%)
Ex-smoker	25685 (12.3%)	520511 (19.2%)	592977 (25.7%)	301162 (28.1%)	169861 (27.9%)

Light smoker	39048 (18.6%)	396915 (14.6%)	276726 (12.0%)	122360 (11.4%)	68982 (11.3%)
Moderate smoker	7763 (3.7%)	79860 (2.9%)	57133 (2.5%)	26251 (2.4%)	14736 (2.4%)
Heavy smoker	3256 (1.6%)	35252 (1.3%)	29759 (1.3%)	14909 (1.4%)	8575 (1.4%)
Missing	9939 (4.7%)	21224 (0.8%)	9808 (0.4%)	4026 (0.4%)	2596 (0.4%)
**Non-obesity related morbidity**					
Asthma, COPD	37294 (17.8%)	388172 (14.3%)	355495 (15.4%)	192961 (18.0%)	137895 (22.7%)
Autoimmune disease	2044 (1.0%)	25797 (1.0%)	26093 (1.1%)	14838 (1.4%)	9707 (1.6%)
Ulcerative colitis, Crohn’s disease	2147 (1.0%)	27626 (1.0%)	24211 (1.0%)	10401 (1.0%)	5388 (0.9%)
Type 1 diabetes	770 (0.4%)	15344 (0.6%)	15569 (0.7%)	8101 (0.8%)	4464 (0.7%)
Chronic liver disease	1237 (0.6%)	13015 (0.5%)	14658 (0.6%)	9727 (0.9%)	6029 (1.0%)
Chronic renal disease	5896 (2.8%)	88120 (3.2%)	122569 (5.3%)	70310 (6.6%)	40268 (6.6%)
Chronic neurological disease	10599 (5.1%)	87922 (3.2%)	76349 (3.3%)	36795 (3.4%)	20653 (3.4%)
Cerebral palsy	655 (0.3%)	2671 (0.1%)	1780 (0.1%)	942 (0.1%)	630 (0.1%)
**Obesity related morbidity**					
Hypertension	14586 (7.0%)	297058 (10.9%)	504164 (21.9%)	329420 (30.7%)	217386 (35.7%)
CVD	7064 (3.4%)	111788 (4.1%)	161851 (7.0%)	89275 (8.3%)	45816 (7.5%)
Sleep apnoea	424 (0.2%)	8155 (0.3%)	22221 (1.0%)	25232 (2.4%)	35727 (5.9%)
GORD	6570 (3.1%)	107232 (4.0%)	135576 (5.9%)	74866 (7.0%)	44245 (7.3%)
Congestive Heart Failure	1888 (0.9%)	23741 (0.9%)	32289 (1.4%)	20919 (1.9%)	14854 (2.4%)
Stroke	3876 (1.9%)	52392 (1.9%)	64133 (2.8%)	32925 (3.1%)	16868 (2.8%)

*Asian includes Indian, Pakistani, Bangladeshi, Other Asian; Black includes Black African.
**Townsend scores were obtained for each patient as a proxy for socioeconomic status. Townsend scores are comparable with the index of multiple deprivation.

Supplemental Table 2. Attributable risks and fractions in risk of severe COVID-19 outcomes associated with BMI across age groups

In this analysis, we calculated the incidence of outcomes in the group who were aged ≥80 years of age and had a BMI of 23-23.9kg/m^2. Severe COVID-19 is relatively markedly more common in older people. For this, we calculated the person-days of exposure and divided the incident cases of our outcomes (hospitalisation, ICU admission, and death) by the person-days of follow up. However, calculating the incidence rates in people with this particular BMI in younger age groups where outcomes were rare would be affected greatly by imprecision. We therefore calculated a modelled or expected incidence by applying the hazard ratio for age to the median age in each age group to the observed incidence in the population over 80 years. This gave us the expected incidence at the median age of each age group in people with a BMI of 23. Thereafter, we applied the hazard ratio for BMI for the BMIs shown in Table 3 (HR constant with age) or Figure 2 (HR differs by age) to the expected incidence of outcomes in each age group. The difference in modelled incidence rates between a BMI of 30, 35, or 40 and one of 23 is the attributable risk, interpreted as the number of additional cases/10,000 persons of severe COVID-19 in each age group at each BMI level. The attributable fraction is the difference in incidence between the exposed (people with BMI of 30, 35, or 40) and the unexposed (people with a BMI of 23) divided by the incidence in the exposed, and can be interpreted as the proportion of cases arising as a result of the exposure (BMI level). As an example, if we accept BMI has a causal association, 77% of the risk of needing hospitalisation for severe COVID-19 in people of 20-39 years with a BMI of 40 can be explained by BMI.

		Attributable risks per 10,000 person-year				Attributable fractions % per 10,000 person-year			
COVID-19 Hospital admission	Incidence rate per 10,000 population	BMI 23 kg/m^2	BMI 30 kg/m^2	BMI 35 kg/m^2	BMI 40 kg/m^2	BMI 23 kg/m^2	BMI 30 kg/m^2	BMI 35 kg/m^2	BMI 40 kg/m^2
20-39 years	0.040	0	21	46	85	0	45	64	77
40-59 years	0.120	0	49	105	187	0	42	60	73
60-79 years	0.316	0	59	113	178	0	24	38	49
80+ years	1.141	0	29	50	73	0	7	11	16
COVID-19 ICU admission									
20-39 years	0.005	0	2	6	12	0	57	77	87
40-59 years	0.025	0	3	8	16	0	52	71	83
60-79 years	0.049	0	2	5	8	0	29	44	56
80+ years	0.025	0	5	10	16	0	33	50	63
COVID-19 Death									
20-39 years	0.001	0	3	9	21	0	67	85	93
40-59 years	0.016	0	14	35	73	0	57	77	87
60-79 years	0.121	0	16	30	46	0	19	30	39
80+ years	0.765	0	0	0	0	0	0	0	0

Supplementary Figure 2. Associations of BMI with severe COVID-19 outcomes: sensitivity analysis using BMI measurements taken over the previous year (n=2,291,940)

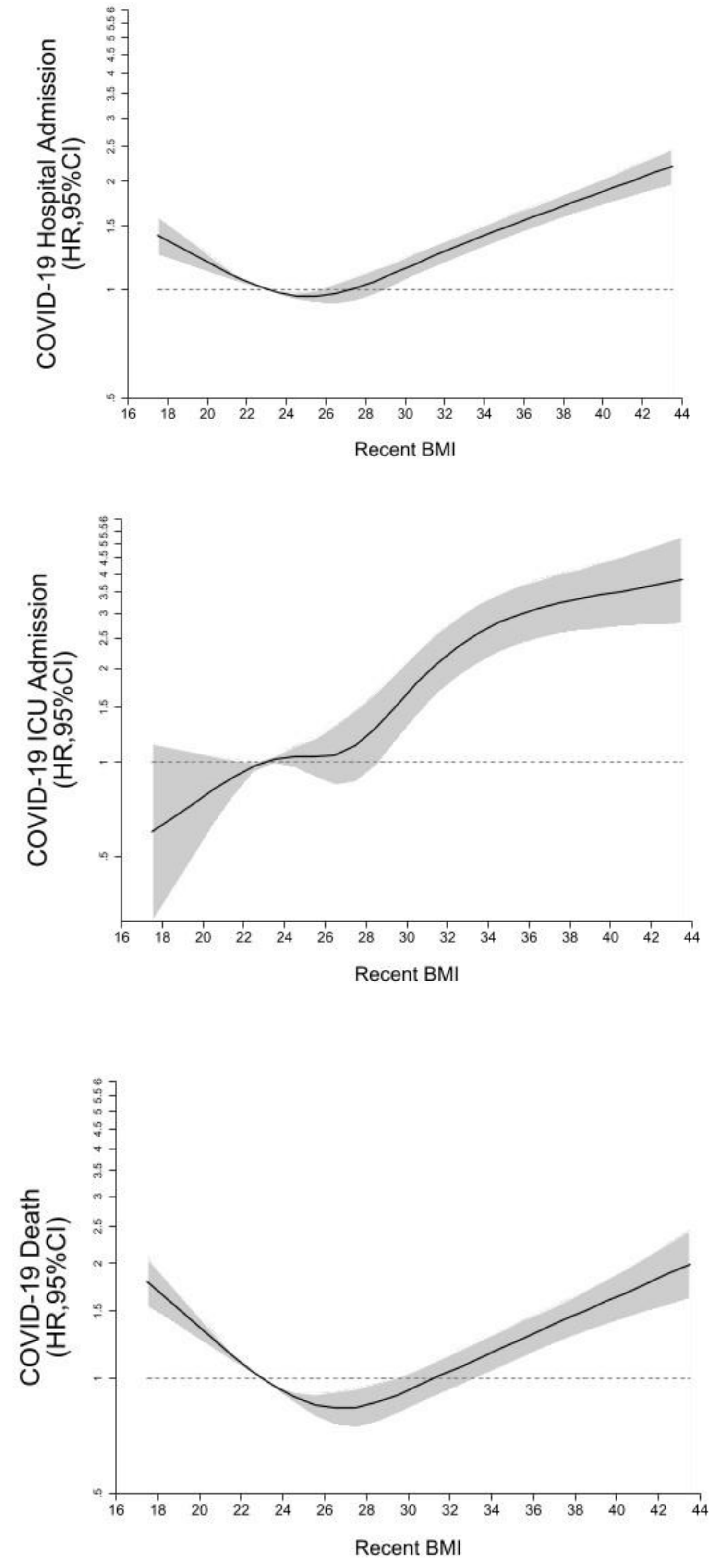

Supplemental Table 3. Sensitivity analyses of BMI with severe COVID-19 outcomes excluding people with recorded BMI before 24th January 2019, excluding people living in care homes and excluding people with missing covariates

Outcomes	Recent BMI measurement N=2,291,940		Excluding care home patients N=6,865,469		Excluding patients with missing data N=5,842,989	
	Cases	HR (95% CI)	Cases	HR (95% CI)	Cases	HR (95% CI)
Hospital admission	6,544	1.05 (1.04 to 1.05)	12,384	1.05 (1.05 to 1.06)	11,764	1.05 (1.05 to 1.05)
ICU admission	780	1.08 (1.07 to 1.10)	1,580	1.10 (1.09 to 1.11)	1,407	1.09 (1.08 to 1.10)
Death	2,764	1.06 (1.04 to 1.07)	4,116	1.06 (1.05 to 1.06)	4,703	1.04 (1.04 to 1.05)

* For the analyses of recent BMI measurements, BMI restricted to nadir, found at BMI ≥25 for hospital admission, and BMI≥28 for death
**For exclusion of patients with missing covariates, people missing ethnicity, Townsend index and smoking were excluded

Supplemental Table 4. Baseline characteristics in people with vs without BMI recorded

	Missing BMI	Not missing BMI
**N**	**1,228,967**	**6,910,695**
Prediabetes	20843 (1.7%)	669846 (9.7%)
Type 2 diabetes	11700 (1.0%)	577246 (8.4%)
**Age group**		
20-39	825116 (67.1%)	2384223 (34.5%)
40-59	277557 (22.6%)	2444011 (35.4%)
60-79	100780 (8.2%)	1652615 (23.9%)
80+	25514 (2.1%)	429846 (6.2%)
**Gender**		
Men	810088 (65.9%)	3242387 (46.9%)
**Ethnicity**		
White	534375 (43.5%)	4771657 (69.0%)
Asian	71822 (5.8%)	550618 (8.0%)
Black	32625 (2.7%)	254507 (3.7%)
Chinese	16476 (1.3%)	64984 (0.9%)
Others/Not recorded	573669 (46.7%)	1268929 (18.4%)
**Townsend deprivation**		
Quintile 1 (most affluent)	239935 (19.5%)	1617387 (23.4%)
Quintile 2	260228 (21.2%)	1536385 (22.2%)
Quintile 3	253970 (20.7%)	1353591 (19.6%)
Quintile 4	236990 (19.3%)	1224177 (17.7%)
Quintile 5 (most deprived)	230245 (18.7%)	1149438 (16.6%)
**Smoking**		
Non smoker	590633 (48.1%)	4071381 (58.9%)
Ex-smoker	135724 (11.0%)	1610196 (23.3%)
Light smoker	163217 (13.3%)	904031 (13.1%)
Moderate smoker	36570 (3.0%)	185743 (2.7%)
Heavy smoker	14043 (1.1%)	91751 (1.3%)
Missing	288780 (23.5%)	47593 (0.7%)
**Non-obesity related morbidity**		
Asthma, COPD	95420 (7.8%)	1111817 (16.1%)
Chronic renal disease	9603 (0.8%)	327163 (4.7%)
**Obesity related morbidity**		
Hypertension	45073 (3.7%)	1362614 (19.7%)
CVD	14541 (1.2%)	415794 (6.0%)

Supplemental Table 5. Risk of severe COVID-19 outcomes in people with vs without BMI recorded

COVID-19 Outcomes	HR (95% CI)
Hospital admission	
Not missing BMI	1 (ref)
Missing BMI	1.01 (0.93 to 1.10)
ICU admission	
Not missing BMI	1 (ref)
Missing BMI	0.70 (0.56 to 0.88)
Death	
Not missing BMI	1 (ref)
Missing BMI	1.38 (1.20 to 1.59)
